# Myelin Oligodendrocyte Glycoprotein Antibody-Associated Disease: A Case Report

**DOI:** 10.7759/cureus.55652

**Published:** 2024-03-06

**Authors:** Smaran S Teru, Jaswanthi Dogiparthi, Thomas J Bonitz, Chris Buzas

**Affiliations:** 1 Medical School, Lake Erie College of Osteopathic Medicine, Erie, USA; 2 Ophthalmology, Lake Erie College of Osteopathic Medicine, Erie, USA

**Keywords:** myelin oligodendrocyte glycoprotein antibody-associated disease, multiple sclerosis, idiopathic intracranial hypertension (iih), myelin oligodendrocyte glycoprotein (mog) antibodies, myelin oligodendrocyte glycoprotein (mog), mogad

## Abstract

Myelin oligodendrocyte glycoprotein antibody-associated disease (MOGAD) is a newly discovered autoimmune demyelinating disorder. The clinical manifestations of MOGAD are divergent but often characterized by inflammatory central nervous system (CNS) deficits such as optic neuritis, encephalitis, or transverse myelitis that predominantly affect the pediatric population. Despite the distinct features often associated with MOGAD, the disease exhibits a diverse range of clinical manifestations, making timely diagnosis and treatment challenging. In particular, distinguishing MOGAD from multiple sclerosis (MS) is important for adequate treatment and the prevention of relapsing disease. In this report, we present a rare case of MOGAD in a 57-year-old male who initially exhibited symptoms of bilateral optic nerve edema and flame hemorrhage. This led to an initial misdiagnosis of pseudotumor cerebri. Serological analysis at a tertiary care center ultimately led to the diagnosis of MOGAD after multiple visits to the ophthalmologist with worsening vision deficits.

## Introduction

Over the past 15 years, the increased identification of various demyelinating central nervous system (CNS) diseases, apart from multiple sclerosis (MS), has paved the path to increased accuracy in the diagnosis of chronic inflammatory CNS conditions. Of the antibody-associated diseases discovered, myelin oligodendrocyte glycoprotein antibody-associated disease (MOGAD) has demonstrated an increasing incidence. Over time, the presence of myelin oligodendrocyte glycoprotein (MOG)-autoantibodies in serum has grown to include various unique presentations, ranging from optic neuritis to encephalomyelitis [1]. The most common clinical manifestation of MOGAD is MOGAD-associated encephalitis, with optic neuritis being the second [2]. Of note, optic neuritis in MOGAD differs from MS as it tends to be bilateral and leads to severe visual deficits if left untreated [2].

Myelin oligodendrocyte glycoprotein is a protein expressed on the external surface of myelin and the plasma membranes of oligodendrocytes. The protein is thought to act as an antigen target for autoantibodies and an autoantigen for demyelination in the CNS, as MOG’s highest density is localized in the outermost lamellae of myelin sheaths [3]. The characterization of the unique clinical, radiological, and therapeutic profiles of patients with MOGAD has developed over the years with advances in diagnosis and management. As new clinical features of the disease become apparent, there is a need to better understand this relatively new pathology. Here, we introduce an extremely rare case of MOGAD in a 57-year-old male uniquely presenting with bilateral papilledema concerning intracranial hypertension. This case adds to the growing clinical evidence of a range of presentations of MOGAD, allowing better clinical decision-making. Increased awareness of the presentation of MOGAD, similar to idiopathic intracranial hypertension, may aid in decreasing misdiagnosis and delays in treatment, which lead to worse clinical outcomes. 

## Case presentation

A 57-year-old male with a medical history of hypertension, factor V Leiden, chronic alcohol abuse, and a recent COVID-19 infection presented to the ophthalmologist's office for a same-day appointment with the chief complaint of vision loss in his left eye (OS) that had been progressively worsening in the past four months. He reported cloudy vision, light sensitivity, and pain around the left orbit but denied any flashes, new floaters, diplopia, photophobia, tearing, redness, headaches, or other related symptoms. His medication list included acetazolamide, sildenafil, atenolol, and chlorthalidone.

Two weeks before this visit, he had visited the emergency department (ED) for persistent vision loss in his left eye. He underwent a lumbar puncture in the ED, revealing an opening pressure of 33 cmH2O. An MRI of his brain and orbits revealed opacification of his left maxillary sinus consistent with his described ear and sinus congestion, productive cough, and nocturnal fevers six to eight weeks prior. He was admitted with the diagnosis of idiopathic intracranial hypertension and treated with IV methylprednisolone and acetazolamide. A repeat lumbar puncture two days later showed an intracranial pressure of 18 cmH2O. He was discharged with 1,000 mg of acetazolamide two times a day (BID) and a three-day prednisone taper, with a follow-up scheduled with neurology.

**Figure 1:**
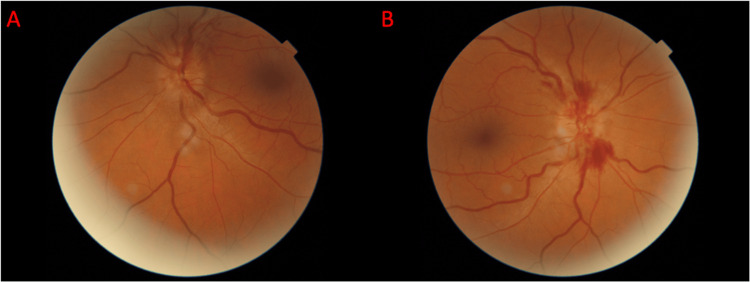
Fundoscopic images were captured of the left eye (A) and right eye (B) from the first visit to the ophthalmologist on May 23, 2023. The findings were notable for 3+ papilledema with retinal nerve fiber layer hemorrhages and cotton wool spots/ischemia in OU and retinal nerve fiber layer hemorrhages in the OD (B) > OS (A). OU: oculus uterque (both eyes); OD: oculus dexter (right eye); OS: oculus sinister (left eye)

In the ophthalmologist's office, visual acuity was limited to 20/150 in the right eye (OD) and hand motion at 3 feet in the OS. Intraocular pressure (IOP) measurements were 12 mmHg in the OD and 8 mmHg in the OS. Pupil examination revealed round pupils in the OD reacting appropriately to light and a 2+ afferent pupillary defect (APD) in the OS. Visual acuity was full to finger counting in the OD and confrontation to center in the OS. Optic nerve fundoscopy revealed severe edema in the left eye compared to the right eye, along with retinal nerve fiber layer hemorrhages in both eyes (OU). Examination of the macula indicated nasal edema secondary to the nerve edema in OU, while the foveal reflex remained intact in OU (Figures 1A, 1B). These findings contributed to his diagnostic evaluation, and the patient was advised to change his acetazolamide regimen to 1,500 mg of acetazolamide BID and an additional two-day prednisone taper while monitoring for symptomatic changes.

Six days later, the patient returned to the ophthalmologist with worsening vision in the OS, now unable to perceive light. Visual acuity was determined to be limited to hand motion at 1 foot in the OD and no light perception in the OS. The patient denied any additional symptoms. During the physical examination, IOP measured 15 mmHg in the OD and 14 mmHg in the OS. Pupillary examination indicated dilation and continued APD. Confrontation visual fields revealed a significant superior visual field defect, with constriction observed in all other gaze directions nearly extending to the center in the right eye. Notably, the left eye exhibited no visual acuity. Examination of the optic nerve revealed severe edema in the left eye, along with flame hemorrhage bilaterally and subtle to no improvement of his papilledema from previous fundoscopic images.

Considering the pronounced decline in visual acuity, further exploration of alternative etiologies became necessary. The patient denied symptoms such as jaw claudication, scalp tenderness, or other classic indicators of giant cell arteritis (GCA). As a precaution, the patient was referred to a larger academic center emergency department for a comprehensive evaluation.

An extensive infectious and rheumatologic workup was initiated upon arrival at the ED. An MRI of the brain revealed abnormal signals and enhancement in the bilateral optic nerves, indicative of optic neuritis, new or significantly increased, from his prior hospital admission. Concurrently, an MRI of the spine showed no discernible evidence of demyelinating lesions affecting the spinal cord, and a PET-CT scan yielded no indications of neoplastic activity.

**Figure 2:**
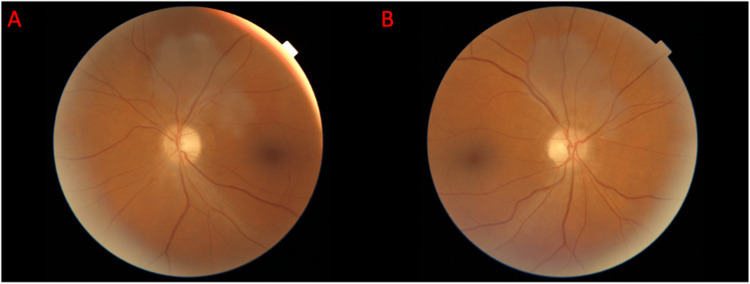
Fundoscopic images captured of the left eye (A) and right eye (B) on a follow-up visit to the ophthalmologist on October 2, 2023, the first visit after completion of the IV steroid regimen, show rapid resolution of the edema with mild residual temporal pallor in OU. OU: oculus uterque (both eyes)

**Figure 3:**
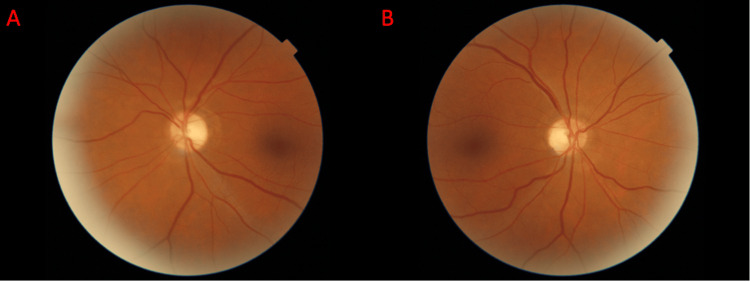
Fundoscopic images captured of the left eye (A) and right eye (B) from the three-month follow-up visit to the ophthalmologist on May 23, 2023, show rapid, near-complete resolution of the edema bilaterally with mild residual temporal pallor in OU. OU: oculus uterque (both eyes)

Antibody screens were conducted, and the patient tested positive for MOG antibody at a titer of 1:1000, substantiating the diagnosis of post-viral autoimmune/inflammatory optic neuritis secondary to MOGAD. This finding was likely superimposed by an underlying optic neuropathy secondary to his alcohol use. Treatment commenced with a five-day, 1,000-mg IV methylprednisolone course and five sessions of plasma exchange therapy. A 60-mg prednisone taper regimen was successfully executed, and the patient exhibited marked clinical improvement in vision. After completing the final plasma exchange session, the patient was discharged and advised to follow up with his primary care provider and neuro-ophthalmology team. The patient’s visual acuity progressively improved at one month (counting fingers at 2 feet in the OD and 20/60 in the OS), six weeks (20/300 in the OD and 20/40 in the OS), and three months (20/50 in the OD and 20/25 in the OS) following discharge. Repeat fundoscopic imaging one week later (Figures 2A, 2B) and three months later (Figures 3A, 3B) procured during the visit to the academic center is shown. 

## Discussion

Myelin oligodendrocyte glycoprotein antibody-associated disease is an inflammatory disease of the central nervous system with a wide range of presenting signs and symptoms [4]. It is characterized by immune-mediated demyelinating attacks of the CNS, affecting the optic nerves, brain, and spinal cord [5].

Early studies discovered that oligodendrocyte surface proteins, known as MOGs, were not considered antibody targets in MS. Therefore, in 2007, immunoglobulin G (IgG) antibodies binding to MOG were categorized as a separate demyelinating disease [5, 6], now referred to as MOGAD. However, the epidemiology and pathogenesis of MOGAD remain unclear due to MOGAD’s recent discovery in 2007, with available MOG antibody testing occurring years later [7, 8]. Studies in Europe predict an incidence between 1.6 and 3.4 per million person-years [7, 8], with one report estimating rates of 3.0 and 1.3 per million person-years in children and adults, respectively [8]. The median age of onset is typically 20 to 30 years, with half of reported cases occurring in the pediatric population [9-11].

Many clinical features of MOGAD are characteristic but not disease-specific, posing challenges for accurate and timely diagnosis. Delays in diagnosis in patients with persistent MOG-IgG seropositivity potentiate an increased risk of recurrence, necessitating prompt diagnosis for a better prognosis [1]. Common clinical manifestations include unilateral or bilateral optic neuritis [9], with up to 50% of patients experiencing optic neuritis bilaterally [12]. Optic neuritis often presents with varying vision loss and is pathognomonically described as eye pain that worsens with eye movement, sometimes preceding complaints of vision loss [13]. In children, eye pain secondary to optic neuritis can be mistaken for non-specific signs of headache, leading to further diagnostic delay [14]. Furthermore, optic neuritis and optic nerve edema often occur concurrently in patients with MOGAD. An observational case series including 87 patients with MOG-IgG seropositivity and optic neuritis reported that 86% of patients presented with optic disc edema and eye pain [13]. Severe optic disc edema can lead to peripapillary hemorrhages, creating further diagnostic challenges for MOGAD, among other etiologies of hemorrhagic papilledema [13]. The patient’s fundoscopic imaging at the initial evaluation revealed 3+ papilledema in OU, with severe edema on the left greater than the right and retinal nerve fiber layer (RNFL) hemorrhage more pronounced on the right than the left (Figures 1A, 1B). These findings led to the initial diagnosis of papilledema secondary to idiopathic intracranial hypertension. 

However, intracranial hypertension is a rare presentation of MOGAD and has been sparsely reported. Increased cerebrospinal fluid (CSF) in the inflammatory context is more commonly associated with manifestations such as acute disseminated encephalomyelitis or meningoencephalitis [15]. One previously reported potential cause of increased CSF production in MOGAD without evidence of encephalitis might involve low-grade meningeal inflammation producing increased CSF [15]. 

Patients with MOGAD may also exhibit extraocular manifestations. For instance, patients with MOGAD, especially children, may present with acute disseminated encephalomyelitis (ADEM), resulting in encephalopathy not explained by fever, systemic illness, or postictal features [16, 17]. Additionally, MOGAD may display features of transverse myelitis, characterized by isolated spinal cord involvement or combined spinal cord involvement with other areas of the central nervous system [18-20]. These features include paraparesis or quadriparesis with loss of sensation and bladder, bowel, and erectile dysfunction [18-21]. Other neurological features of MOGAD encompass cerebral monofocal or polyfocal deficits, brainstem and cerebellar features, cerebral cortical encephalitis, or seizures [22-25]. However, it is worth noting that none of these features were observed in the case we are presenting.

Given the similarities in clinical presentation, distinguishing MOGAD from MS is crucial for improved long-term management. Although optic neuritis is a non-specific clinical feature in both MOGAD and MS patients, distinctions exist. Vision loss tends to be more central and severe in MOGAD than in MS [13]. Additionally, optic nerve enhancement typically extends over 50% of the nerve's length in MOGAD, in contrast to less than 50% in MS seen in a T1-weighted post-gadolinium brain MRI [13].

To aid in differentiation, diagnostic measures go beyond a qualitative assessment of presenting features. For instance, MOG-IgG at higher titers can be instrumental in distinguishing MOGAD from MS [26]. Notably, testing for serum MOG-IgG antibodies with a cell-based assay may carry the potential for false-positive results [27]. In a study involving 2,107 adult inpatients in a German hospital evaluated for various neurological diseases, 1.2% were found to be MOG-IgG positive at low titer, but only 0.2% had true MOGAD [27]. Therefore, a higher titer cell-based assay is the preferred method for distinguishing MOGAD from MS, in conjunction with sound clinical judgment. Furthermore, CSF oligoclonal bands are typically present in most MS patients and can assist in discriminating MOGAD from MS. They are found in 5% to 20% of MOGAD patients and 88% of MS patients, respectively [28-31]. 

Given the acuity of the patient’s visual deficits and MRI findings supporting bilateral optic neuritis at the tertiary center, the patient most likely experienced an acute flare of MOGAD in the setting of sinusitis and a systemic inflammatory response. The patient’s five-day course of 1,000 mg intravenous methylprednisolone and a 60 mg prednisone taper mirror supporting literature for the treatment of acute attacks. Myelin oligodendrocyte glycoprotein antibody-associated disease (and MS) tends to respond appropriately to glucocorticoid therapy [32]. The recommended acute treatment for both inflammatory diseases is a three-to-seven-day course of 500 mg or 1,000 mg intravenous methylprednisone with or without a prednisone taper and plasma exchange therapy for patients refractory to glucocorticoid therapy [33-34]. Successful long-term prevention of relapsing disease in MOGAD includes rituximab, mycophenolate mofetil, intravenous immunoglobulin (IVIG), and azathioprine, but additional research is required to assess relapse prevention [10, 35-37].

## Conclusions

We present a unique case of MOGAD with rare manifestations of the demyelinating autoimmune disorder to increase our understanding of the disease and recognize its broad clinical presentation, including increased intracranial hypertension. Although our patient presented with optic neuritis, a common presentation of MOGAD, the unusual presentation of increased intracranial hypertension with bilateral papilledema caused a misdiagnosis of the patient’s symptoms, widening the possibility of ophthalmic manifestations of MOGAD. We aim to promote the accurate diagnosis of MOGAD in the setting of an unusual presentation to increase the likelihood of raised suspicion and prompt treatment after appropriate serological studies while adding to existing literature to guide future management. 
